# Thyroid Dysfunction Prevalence and Risk Factors in the Southeastern Part of Bangladesh: A Cross‐Sectional Study

**DOI:** 10.1002/hsr2.70329

**Published:** 2025-01-07

**Authors:** Muhammad Shahadat Hossen, Md. Mahmodul Islam, Abhijit Das, Md. Abdur Rahman Ripon, Mohammad Tohidul Amin, Mohammad Anwarul Basher, Md. Mamun Or Rashid

**Affiliations:** ^1^ Department of Pharmacy Noakhali Science and Technology University Noakhali Bangladesh

**Keywords:** Bangladesh, dietary supplement, hyperthyroidism, hypothyroidism, iodine therapy, thyroid disorder

## Abstract

**Background and Aims:**

Globally thyroid disorders (TDs) are common health issues including in the Southeast Asian region. The purpose of this study was to ascertain the prevalence of thyroid disorders in the Bangladeshi population, with a particular focus on the frequency, etiology, and comorbidity of various thyroid diseases.

**Methods:**

A cross‐sectional research design was conducted in the southeastern part of Bangladesh. Information regarding prior diagnosis and current management of TDs was obtained through a questionnaire.

**Results:**

Of the 300 respondents, 209 were female, and 91 were male. Females were found to be more predominant across all categories of TDs (*χ* ^2^ = 17.738, *p* < 0.05). Furthermore, the older age group (31–45 years) demonstrated a higher frequency of thyroid problems (OR: 1.320). Specifically, hyperthyroidism was identified as the most common thyroid disorder, followed by hypothyroidism, and euthyroidism. Hyperthyroidism was found to be less common in males (18.69%) compared to females (43.06%), while hypothyroidism was more prevalent in males (46.15%) compared to females (27.75%). Multiple logistic regression analysis demonstrates that participants with a family history of TDs have a significantly higher chance (OR: 2.991, CI: 1.480–6.044, *p* < 0.05) of having hyperthyroidism than those without such a history. Interestingly, the studied population also exhibited higher rates of comorbidities including gastritis, diabetes, and cardiovascular diseases.

**Conclusion:**

According to the study's findings, it is advised to concentrate on educating Bangladeshis about thyroid problems, particularly female population. Moreover, healthcare professionals should check elderly patient's thyroid conditions and consider the possibility that thyroid dysfunction can coexist with other conditions.

## Summary

- The rate at which thyroid disorders are affecting people worldwide at any age is concerning.
- The family history of hyperthyroidism significantly contributed to the development of such diseases. Additionally, this disease aided in the emergence of conditions similar to diabetes, heart disease, and gastritis.
- Reduced thyroid‐related morbidity can improve the quality of life for those affected while also contributing to the achievement of broader global health goals.

**Abbreviations**
AITD: autoimmune thyroid diseases
CI: confidence intervals
E: east
FT_3_
: free triiodothyronine
FT_4_
: free thyroxine
N: north
NCDs: noncommunicable disease
OPD: out‐patient‐department
OR: odds ratio
SDGs: sustainable development goals
SPSS: statistical package for the social sciences
SE: standard error
TBG: thyroxine‐binding globulin
TBPA: transthyretin (TTR)/pre‐albumin
TDs: thyroid disorder
TSH: thyroid stimulating hormone
TT_3_
: total triiodothyronine
TT_4_
: total thyroxine

## 1 Introduction

Thyroid disorders (TDs) are common endocrine disorders with variable prevalence among the population of the whole world [1]. Located in the lower neck, the thyroid gland is a butterfly‐shaped tissue [2]. Growth, brain development, and the intensity of chemical reactions in our bodies are all regulated by the production of hormones containing iodine [3, 4]. Supporting that, thyroid gland disorder is more prevalent than other diseases those are not communicable [5]. Common thyroid problems include hypothyroidism which is characterized by low functional thyroid hormone levels, and hyperthyroidism which is associated with high functional thyroid hormone levels. In addition, some patients also observe structural abnormalities and enlargement of the thyroid glands, tumors benign or carcinogenic tumors [6, 7]. Iodine shortage, inflammation, autoimmune diseases, nodules or noncancerous lumps, malignant tumors, and certain medical treatments, such as radiation therapy or thyroid surgery and so forth can also contribute to develop TDs [8, 9]. Hypothyroidism commonly manifests as weakness, low energy, weight gain, cold sensitivity, bradycardia, dry skin, and constipation [10]. Sometimes a part of the neck may be swelling which is called as goiter [11]. However, minor changes in thyroid function could be related to issues with weight gain and blood pressure [12, 13].

Thyroid diseases (TDs) are most prevalent endocrine condition globally including Southeast Asia. Over 1 billion people live in iodine‐deficient areas ranging from Southeast Asia and South America to Central Africa [14]. According to estimates, among men, 1 in 10 people has thyroid illness; and this ratio is 1 in 8 for women. A 50% undiagnosed or incorrectly diagnosed thyroid condition is thought to exist. Although it is not directly considered a noncommunicable disease (NCDs), it is well believed that TDs are the precursors of many NCDs, like diabetes mellitus, high blood pressure, cancer and so forth [5].

The precise prevalence of thyroid disease in Bangladesh remains mostly undisclosed. It is widely acknowledged that the prevalence of thyroid diseases in Bangladesh ranges from approximately 10% to 20% among the general population [15]. Despite a drop in iodine‐deficient illnesses since the early 1990s, the prevalence and variety of thyroid disorders in Bangladesh remain inadequately documented due to a lack of published reports. A study published in 1995 documented that 35% of all thyroid diseases were attributed to iodine deficiency as the primary cause. The remaining cases consisted of autoimmune conditions (26%), malignant tumors (2.58%), and various other thyroid illnesses [16]. In Bangladesh, during the early years of the twenty‐first century, prevalent thyroid illnesses include hypothyroidism, Graves' disease, postpartum thyroiditis, and thyroid cancers. However, it is worth noting that iodine deficiency disorders continue to affect individuals at a low prevalence rate [17].

Over the past few years, several small‐scale investigations undertaken in various regions of Bangladesh have provided evidence regarding the prevalence of hypothyroidism and hyperthyroidism. Specifically, this research revealed that the prevalence of hypothyroidism was 4.97% and 7.0% in the Khulna and Dhaka districts, respectively. Additionally, the prevalence of hyperthyroidism was found to be 0.86% and 1.04% in the same districts, respectively [15, 18]. Still, there remains a scarcity of published literature regarding the range of thyroid disorders in Bangladesh, particularly among the elderly population residing in the southern region of the country. Considering the severity of thyroid disease in Bangladesh, this cross‐sectional survey‐based study was designed to assess the prevalence of TDs, its linkage with other associated diseases, and concurrent treatment practices in the southeastern part of Bangladesh.

## 2 Materials and Methods

### 2.1 Study Design

This cross‐sectional study aims to investigate the prevalence of thyroid dysfunction and identify associated risk factors among the population residing in the southeastern region of Bangladesh. The study employed a structured questionnaire administered through face‐to‐face interviews to gather demographic information, medical history, and lifestyle factors from participants. A systematic random sampling method was used to select patients from Out‐patient‐department (OPD) across the sites of the study. Data analysis involved descriptive statistics to report the prevalence of thyroid dysfunction categorized by age, gender, and geographical location. Multivariate logistic regression was employed to assess the association between potential risk factors (such as age, gender, iodine intake, family history of thyroid disease, and socioeconomic status) and the prevalence of thyroid dysfunction [19].

### 2.2 Study Setting

The study was conducted from July 2021 to February 2022 and the Chittagong Medical College and Hospital, Chittagong (Geographic coordinates: 22.36° N, 91.78° E) and Comilla Medical College and Hospital, Comilla, Bangladesh (Geographic coordinates: 23.32° N, 90.43° E) served as the site of this study, as they both served enriched Nuclear Medicine and Allied Sciences Department, where TDs were treated.

### 2.3 Study Populations

After conducting initial interviews with 550 participants, a total of 300 people were added to the study, considering variables including gender, age, and disease severity. The sample size was calculated using the following formula, considering the 40% prevalence of TDs with an age > 18 in Bangladesh [15], 95% confidence intervals (CI), and 5% precision values [20]. Where n is the sample size, *z* is the statistic corresponding to the level of confidence, *P* is expected prevalence, and d is precision (corresponding to effect size).

$$n=\frac{P\left(1-P\right)z^{2}}{d^{2}}.$$

### 2.4 Inclusion and Exclusion Criteria

Age > 18 years with thyroid dysfunctions were inclusion criteria for the sample subjects; non‐cooperation, prior surgical history, chronic inflammatory diseases, Class I and V congestive heart failure, blood pressure > 180 mmHg (systolic) or > 110 mmHg (diastolic), chronic liver disease, and pregnancy were the exclusion criteria.

### 2.5 Ethical Statement

The institutional review board's guidelines for working with human subjects were followed in obtaining the subject's informed consent. The ethical review committee of the Noakhali Science and Technology University (NSTU) granted permission to perform this research (approval number is NSTU/SCI/EC/2023/211). The ethical requirements of the institutional and national research committees, as well as the 1964 Helsinki Declaration, its later update, or comparable ethical principles, were upheld in all the strategies [21].

### 2.6 Study Tools

**Figure 1:**
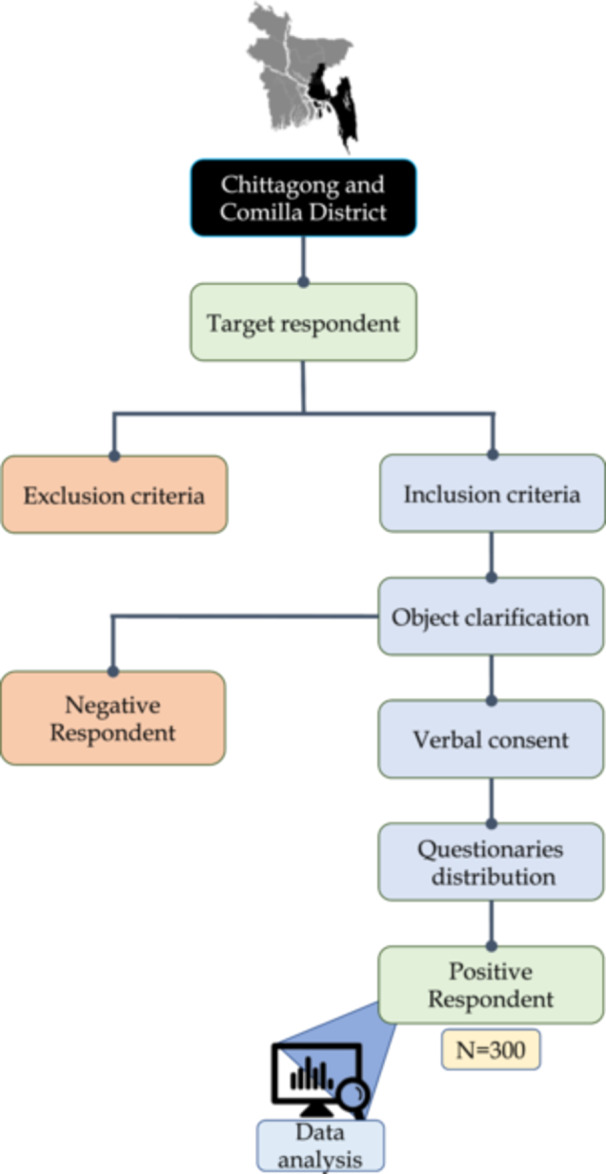
Flowchart of the study.

The primary tool for data collection is a structured questionnaire designed to gather demographic details, medical history, lifestyle habits, and information on potential risk factors such as dietary habits and exposure to environmental factors. An interviewer from each informant ensured verbal agreement after thoroughly outlining the study's goals. This was accomplished by using open‐ended questions. English was used to write the questionnaires, but the native language was used to conduct the interviews (Bengali). For better information, patients were asked to present their prescriptions and the results of any medical tests, as well as to freely complete the survey. The question “Do you have a history of thyroid disorder?” served as the dependent variable in the current research. There were two sections of the record questionnaires used. One part addressed their personal information, which included name, age, gender, weight, and location, while the other part collected their medical condition, like the information in Supporting Information S1: Table S1. Figure 1 shows the entire data‐collecting process, which includes the genetic data, feminine complexity, comorbidities, and diagnosis characteristics.

### 2.7 Statistical Analysis

The raw data was preprocessed in Microsoft Excel (Microsoft Corporation. Office 365: Microsoft Excel; Retrieved from https://office.microsoft.com/excel) before being analyzed statistically. Exploratory data analysis was used on the original data set to look at outliers and missing data completely. The information was then coded and entered SPSS 27.0 (IBM Corp. Released 2020. IBM SPSS Statistics for Windows, Version 27.0. Armonk, NY: IBM Corp Statistical Package for the Social Sciences) for further analysis. The study employed bivariate analysis to examine the correlation of thyroid disorder with gender. This analysis involved the utilization of cross‐tabulation and Pearson's Chi‐square test (χ^2^) to discover any statistically significant connection between the variables (*p* < 0.05 was statistically significant). Following this, a statistical study was performed using multinomial logistic regression to ascertain the impact of the independent variables on the probability of developing thyroid disorders. The Goodness of Fit Test developed by Hosmer and Lemeshow was employed to assess the appropriateness of the binary logistic regression model in accurately representing the data set. A larger *p*‐value indicated that the model was suitably fitted [22].

## 3 Results

### 3.1 Prevalence of Thyroid Disorders Based on Gender Differences

This paper‐based survey was completed by 300 participants in total. More than two‐thirds (69.67%) of the respondents (*n* = 209) were female, while less than one‐third (30.33%) were male (*n* = 91). Thyroid disorders (TDs) exhibited a higher prevalence among women, as seen by a female‐to‐male ratio of 2.3:1. Table 1 presents the distribution of various thyroid disorders across the examined population. The data reveals a statistically significant association between gender and the prevalence of thyroid disorders (*χ* ^2^ = 17.738, *p* < 0.05). The findings of our study indicate that females are significantly at more risk (OR = 2.064; 95% CI = 1.072–3.977; *p* < 0.05) of developing hyperthyroidism compared to male respondents. In contrast, the prevalence of hypothyroidism is lower among female respondents compared to males, as indicated by an odds ratio of 0.511 (95% CI = 0.244–1.069).

**Table 1:** Cross‐tabulation and Chi‐square test of association between thyroid disorders and gender differences.

Variables	Categories	Sex		Chi‐square	*p* value
		Female (*N* = 209)	Yes (*N* = 91)		
**Thyroid disorders**	Euthyroid	61 (20.33%)	32 (10.67%)	—	—
	Hypothyroid	58 (19.33%)	42 (14.00%)	17.738	0.000
	Hyperthyroid	90 (30.00%)	17 (5.67%)	—	—

*Note: p* < 0.05 is considered statistically significant.

### 3.2 Factors Associated With Thyroid Disorders Among Participants

**Figure 2:**
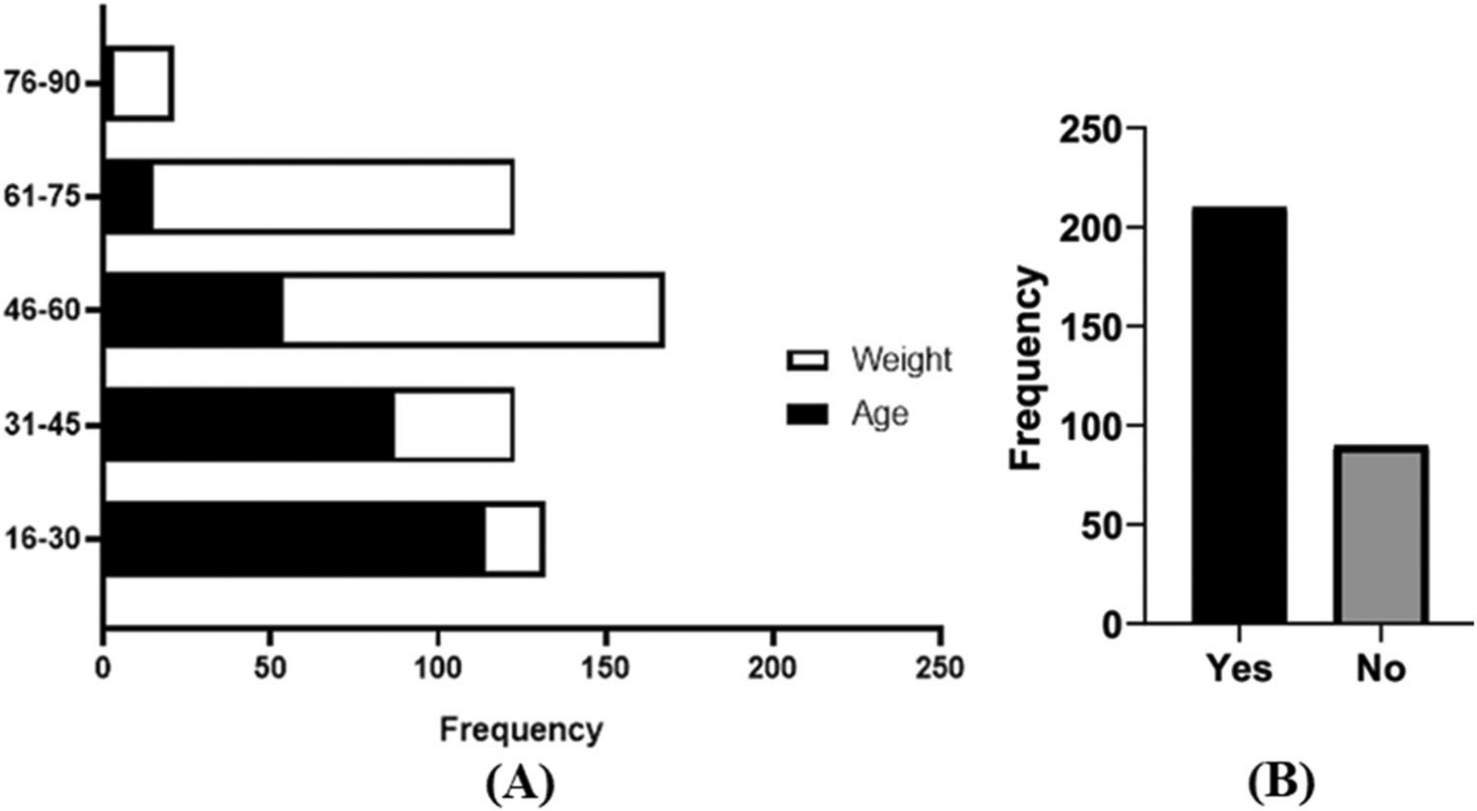
Prevalence of TDs. (A) Based on age and weight variation. (B) Genetic factors (heriditical linkage).

Table 2 displays the outcomes of multinomial logistic regression analysis, utilized as a method of multivariate statistical analysis, to determine the variables that exhibit a significant (*p* < 0.05) impact on thyroid disorders among the participants. Age is an essential factor influencing thyroid function [23]. According to our survey study, at the age of 16–30 years, the prevalence of TDs was the highest (46.67% of the studied population). The odds ratio of 1.320 suggested that participants in the age category of 31–45 years, had a 32% higher likelihood of experiencing thyroid disorders compared to participants in the 16–30 age group. In contrast, the odds ratios of 0.846, 0.970, and 0.72 indicate that individuals in the age groups of 46–60, 61–75, and 76–90 years, respectively, had a reduced probability of 15.4%, 3%, and 28% in developing thyroid disorders in comparison to the participants in the 16–30 age group (Figure 2A).

**Table 2:** Multinomial logistic regression determines the influential factors of thyroid disorders among the respondents.

Variables	Categories	*B*	SE	Exp (*B*)	95% CI for Exp (*B*)		*p* value
					Lower	Upper	
**Age (years)**	16–30	—	—	—	—	—	—
	31–45	0.278	0.314	1.320	0.714	2.441	0.376
	46–60	−0.168	0.383	0.846	0.399	1.791	0.662
	61–75	−0.031	0.655	0.970	0.269	3.500	0.963
	76–90	−0.327	1.203	0.721	0.068	7.623	0.786
**Weight (kg)**	16–30	—	—	—	—	—	—
	31–45	0.811	0.619	2.251	0.669	7.571	0.190
	46–60	0.628	0.541	1.874	0.649	5.413	0.246
	61–75	0.155	0.544	1.167	0.402	3.387	0.776
	76–90	0.508	0.722	1.662	0.403	6.843	0.482
**Family history of thyroid disorder**	No	—	—	—	—	—	—
	Yes	1.096	0.359	2.991	1.480	6.044	0.002
**Hosmer and Lemeshow goodness of fit test**		Chi‐square = 11.499			*p* = 0.175		

*Note: p* < 0.05 is considered statistically significant. SE denotes standard error; CI denotes confidence interval. The Hosmer‐Lemeshow goodness of fit test was conducted to assess the appropriateness of the binary logistic regression model in fitting the data. The test generated a Chi‐square statistic of 11.499 and a *p* value of 0.175. These results suggested that the binary logistic regression model is a good fit for the data and produces more suitable outcomes.

In our study, most TD patients exhibit a notable body weight falling within the range of 46–60 kg, which accounts for approximately 38% of the population under study. The frequency of thyroid problems at different weight variations within the study population is depicted in Figure 2A. According to the analysis, the odds ratios of 2.251, 1.874, 1.167, and 1.662 indicate that individuals in the weight categories of 31–45, 46–60, 61–75, and 76–90 kgs, respectively, had a considerably higher probability of encountering thyroid issues in comparison to those in the 16–30 kg weight group.

It is hypothesized that TD can sometimes develop in genetically predisposed individuals after exposure to environmental stimuli. In our survey, we found that females were more prone to be affected genetically than males (*χ* ^2^ = 12.769, *p* < 0.05). Out of 300 patients, only 90 have a family history of thyroid disease. Among them, 4.67% were male, and 25.33% were female. The frequency of genetic factors is shown graphically in Figure 2B. The findings of the study indicated that female participants with a pre‐existing family history of thyroid diseases had a higher susceptibility to experiencing difficulties in the thyroid gland (OR = 2.991, CI = 1.480–6.044, *p* < 0.05) compared to male participants with a similar familial background.

### 3.3 Common Symptoms and Feminine Complications in TDs Patients

**Figure 3:**
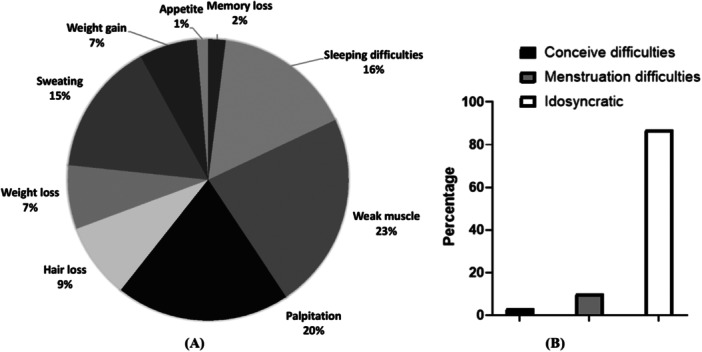
Symptoms and complications of the studied population. (A) Prominent symptoms seen in TDs patients. (B) Complications of the female TDs patients.

Many symptoms were noticed in TDs patients which is presented in Figure 3A. Sleep disturbances, muscle weakness, palpitation, and so forth. were very common among the studied populations. In addition, hair loss, weight gain, weight loss, and so forth. were also noticeable symptoms suffered by TDs patients.

The prevalence of different feminine complexities was found in TDs‐female patients. Our study showed that among the 209 female patients, only 9 respondents had pregnancy or conceiving difficulties due to thyroid disorder, and only 30 faced menstrual cycle difficulties (Figure 3B). Menstrual problem was the most common problem of female TDs patients rather than pregnancy problem during the study (*χ* ^2^ = 12.094; OR: 3.593, CI = 1.675–7.706, *p* < 0.05).

### 3.4 Comorbidities and Thyroid Dysfunctions

**Figure 4:**
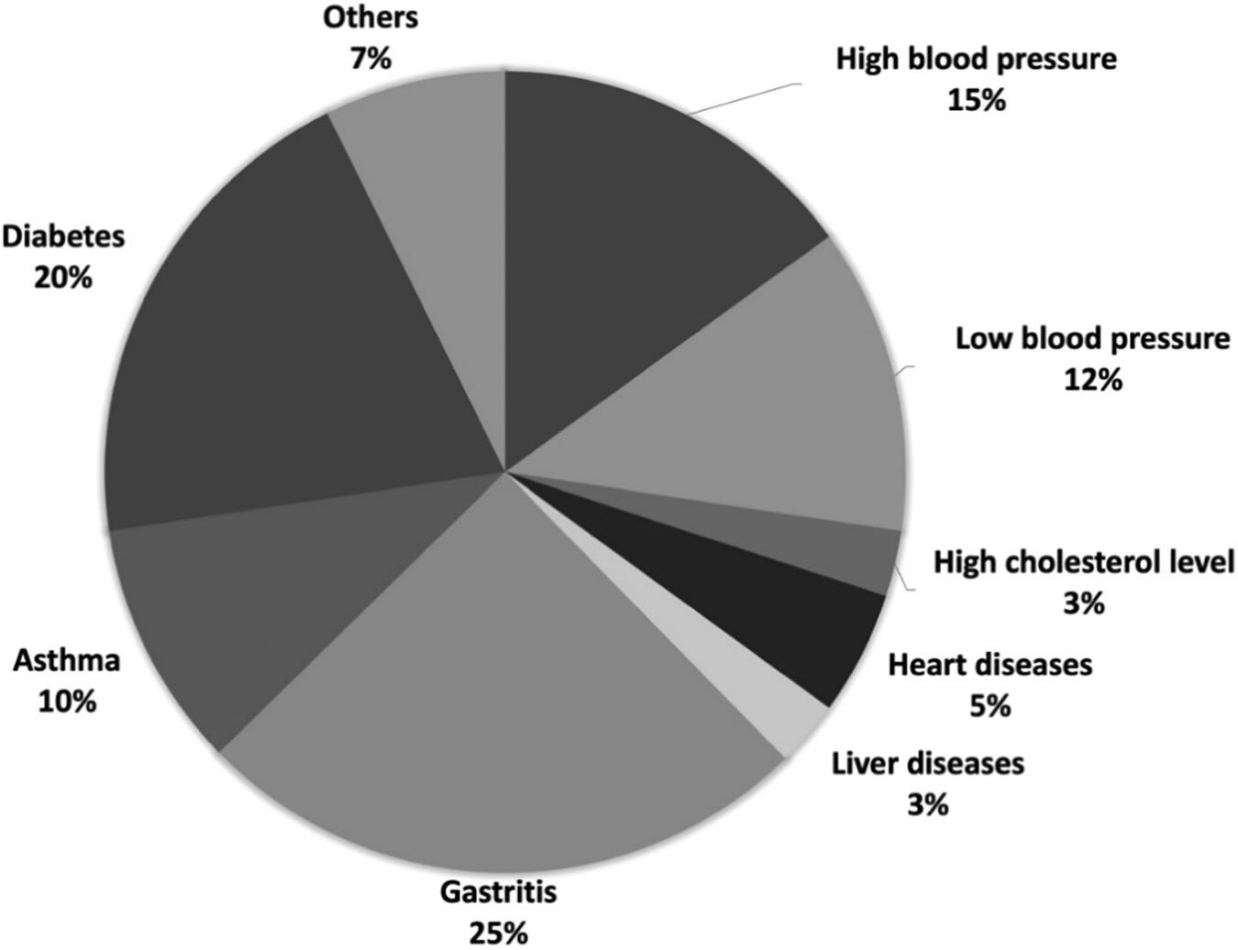
Comorbidities of the TDs patients.

In our study, it was observed that TDs were associated with high blood pressure (*n* = 45), low blood pressure (*n* = 37), high cholesterol level (*n* = 08), heart diseases (*n* = 15), liver diseases (*n* = 08), gastritis problems (*n* = 75), asthma (*n* = 30) and diabetes (*n* = 60). The percentiles of the TDs‐patients suffering from various associated diseases are shown in Figure 4.

### 3.5 Diagnosis Parameters and Treatment Lineup for Thyroid Dysfunctions

Total (TT_4_ and TT_3_), free (FT_4_ and FT_3_), and other serum‐based tests are available by immunoassay to measure the concentration of thyroid hormones in the circulation [24]. Thyroid hormone‐binding plasma proteins, transthyretin (TTR)/pre‐albumin (TBPA), albumin, and thyroxine‐binding globulin (TBG) can all be directly measured. However, the third generation thyroid stimulating hormone (TSH, thyrotropin) assay is the thyroid test measurement with the greatest usefulness for assessing patients suspected of having thyroid illness [25].

**Figure 5:**
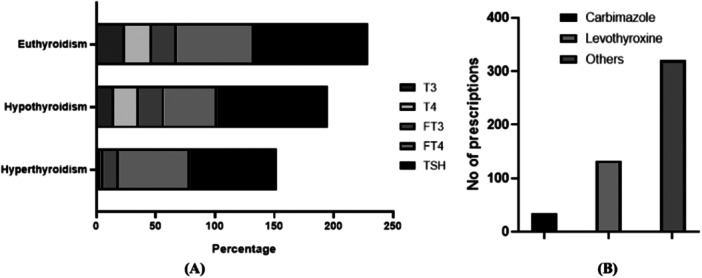
Diagnostic pattern and prescription trend of the studied population. (A) Diagnostic parameters to assess thyroid conditions (B) Prevalence of drugs prescribed in TDs.

In our survey, it was observed that the physicians usually rely on blood sample measurement tests for the detection of thyroid hormone levels; most commonly‐ thyroid stimulating hormone (TSH), free thyroxine (FT_4_), free triiodothyronine (FT_3_), thyroxine (T_4_), triiodothyronine (T_3_), and so forth. Figure 5A describes the overall diagnosis pattern. It was observed that for all types of TDs, FT_4_ and TSH were more common.

Different generic drugs were prescribed to the TDs patients of Bangladesh (Figure 5B). From the prescribed medications, Carbimazole and Levothyroxine were found to be the most frequently used drugs. Along with these medications, both patients were suggested to take Calcium and Vitamin D_3_ tablets as supplementary drugs.

## 4 Discussion

Like other lower‐income nations, the frequency of female health check‐up programs is insufficient in Bangladesh. Thyroid diseases are quite common and affect people of all ages. Targeting this health issue is in line with the Sustainable Development Goals (SDGs) of the World Health Organization, especially Goal 3, which focuses on ensuring good health and well‐being. By focusing on lowering thyroid‐related morbidity, we can enhance the lives of those who are impacted while simultaneously advancing larger global health goals. To lessen the effects of thyroid disorders in Bangladesh and edge closer to SDG 3, it is essential to implement accessible healthcare, spread awareness, and assure prompt diagnosis and treatment. Based on the survey, we found that thyroid disorders (TDs) were more common in women, with a female‐to‐male ratio (2.3:1). Similar observations were reported by some researchers from Nepal, Latin America, and China [26, 27, 28]. A statistically significant association was found between gender and the prevalence of hyperthyroidism. Females were more prone to hyperthyroidism than males. In contrast, this scenario was reversed for hypothyroidism. Males were more susceptible to hypothyroidism than their counterparts. However, there was no significant association found between gender and the prevalence of euthyroidism. The frequencies of thyroid diseases in this sample were like those reported by previous studies in Brazil, Puerto Rico, and Nepal [26, 27, 29, 30]. In our investigation, mild thyroid impairment was usually found. Since most of these individuals had no symptoms, they did not first seek treatment for thyroid issues.

Many factors including ageing, overweight, and genetic factors are responsible for developing TDs [31]. When biological processes deteriorate after an organism reaches its maximum reproductive capacity, it is known as aging. Aging is characterized by a succession of morphological and functional changes throughout time. It is an essential factor influencing thyroid function [23]. Age promotes a natural decrease in pituitary TSH secretion and T4 deiodination, but age also increases the prevalence of anti‐thyroglobulin and anti‐thyroperoxidase antibodies, according to research by Bensenor et al. [23]. Many studies supported the concept that TD is more prone to elderly age [32, 33]. In our study, we also found that almost 53% of the TDs‐patients had an age > 30 years, which implies that aging has a significant effect on the prognosis of TDs. On the other side, the prominence of TD was not identical in any weights. However, the patients ranged in 46–75 kg weight and had the highest (approx. 74%) tendency of thyroid diseases. Figure 2A represents the thyroid prevalence at various ages and weights of the studied population. It implies that gaining body weight may incur TDs‐development. Ylli et al. describe that weight gain and obesity are directly linked with the changes in levels of hormones in the human body which are considered as major reasons for several endocrine diseases [34]. Autoimmunity (Hashimoto's disease), in which the body assaults the thyroid gland, is the most common cause of thyroid function abnormalities. Even, it accounts for more than 90% of non‐iatrogenic hypothyroidism in nations with adequate iodine supplies [35, 36]. An alteration in the immunological tolerance of self‐antigens is the cause of autoimmune illness. It is believed to happen in those with genetic susceptibility after exposure to environmental factors. Nevertheless, it is frequently ignored that gender plays a very important role in the genetic contribution to autoimmunity, with the presence or absence of a Y chromosome increasing the risk for autoimmune disease [37, 38]. It is familiar that most of the TDs originated from autoimmune thyroid diseases (AITD). In this study, we found that females were more prone to TDs and had a higher risk of developing TDs due to familial genetic alteration than males (Figure 2B).

In our study, we found several symptoms of TDs patients which are represented in Figure 3A. Muscle weakness, palpitation, and sleeping difficulties were more common. On the other hand, thyroid hormones interact with sex hormone‐binding globulin and have direct effects on the ovaries, which allows them to play an important part in normal reproductive physiology [39]. We found that after idiosyncratic reasons, menstrual problem was the most common problem of female TDs‐patients rather than pregnancy problems. TDs can lead to menstrual irregularities and infertility. Similar observations were also reported by various studies performed in India and Nepal [40, 41].

According to our study, many diseases were observed concurrently in patients along with their thyroid disorders (Figure 4). Gastritis was the most common comorbid disease for TDs patients (25%). Many researchers reported that thyroid disorders and gastric autoimmune disorders have a positive correlation [42, 43]. This inter‐linkage was first described in the 1960s. The most prevalent autoimmune disease, Hashimoto's thyroiditis, has a known association with gastric disorders in 10‐40% of individuals [35]. The second highest comorbid disease was diabetes which is a common disease for 20% of respondents (Figure 4). Predictably, the TDs patients may suffer from diabetes, because both diabetes and thyroid diseases are caused by endocrine dysfunctions and have a mutual effect on each other's functioning. For TDs patients, type 2 diabetes mellitus is caused by altering the expression of some responsible genes, and physiological abnormalities including a decrease in glucose uptake, an increase in the hepatic glucose clearance, and so forth. In addition, insulin resistance can occur in both hyper and hypothyroidism [44]. Cholesterol (LDL) level was higher for approximately 3% of the TDs‐patients. On the other hand, patients with impaired thyroid function typically have a disrupted lipid profile, which increases their susceptibility to coronary heart disease [45, 46]. Our findings are consistent with this reality. Abnormality of blood pressure and coronary heart diseases were also common comorbidities of the TDs patients. Approximately 32% of TDs‐patients were suffering from the above‐mentioned diseases. Overt and mild thyroid dysfunction both increase the risk of coronary heart disease and mortality, according to Cooper & Biondi [11]. Triiodothyronine (T_3_) can affect the cardiac pacemaker and vascular smooth muscle's functioning in a genomic and non‐genomic manner. Moreover, it can affect myocardial contractibility by acting on myocardial structure and regulatory gene transcriptions [47].

Approximately 40% of the population suffers from thyroid dysfunction in Bangladesh in any part of their life cycle [15]. Bangladeshi physicians relied on blood sample tests for the diagnosis of thyroid disorders. TSH and FT_4_ tests were more common, whereas FT_3_, T_3_, and T_4_ tests were also suggested for the evaluation of the thyroid level in the blood (Figure 5). A higher TSH level confirms hypothyroidism, whereas a lower level of TSH implies hyperthyroidism [48]. Carbimazole was widely used in treating hyperthyroid patients and Thyroxine (Levothyroxine) was extensively used in treating hypothyroidism. One of the key medications listed by the World Health Organization as being necessary for primary healthcare is levothyroxine, which is the cornerstone of the treatment for hypothyroidism [49]. In addition, supplementary drugs like calcium, Vit‐D_3_, Vit‐B_12_, and so forth were also suggested for the TDs‐patients in Bangladesh.

Thyroid diseases have been the subject of few cross‐sectional studies conducted in Bangladesh, especially in areas like Chittagong. Our study clarifies the incidence and consequences of thyroid disorders in this group, which adds important new information to the field of public health. Notwithstanding, it is imperative to recognize the constraints of the research, such as a limited sample size and possible deficiencies in accounting for diverse anthropometric and biochemical factors. The results highlight the need for thorough study to guide targeted interventions and improve healthcare strategies for thyroid diseases in Bangladeshi women, in line with the international commitment to reaching sustainable health goals.

## 5 Recommendation

Considering thyroid diseases in Bangladesh, our study proposes several directions for further investigation and public health campaigns. First, to offer a more thorough picture of the prevalence and factors contributing to thyroid problems in the nation, larger‐scale, longitudinal studies with different populations, encompassing regions beyond Chittagong, are clearly needed. To improve the accuracy and application of results, efforts should also be focused on enlarging sample sizes and incorporating a wider range of anthropometric and biochemical characteristics, to address the limitations of our study.

Campaigns for public health should emphasize the need of routine medical examinations and increase public knowledge of thyroid diseases, particularly in relation to women. Regular thyroid screening should be incorporated into current healthcare initiatives to facilitate early identification and treatment. To guarantee that thyroid diseases can be treated affordably and that diagnostic tests are easily accessible, the healthcare system should also be reinforced. Working together, academics, policymakers, and healthcare professionals can develop evidence‐based policies and interventions that are tailored to the problems that thyroid disorders present in Bangladesh. In the education field, adding details regarding thyroid health to curriculum and community outreach initiatives might enable people to identify symptoms early and seek prompt medical assistance. Comprehensive prevention measures for thyroid health should be developed by research projects that examine the effects of environmental factors, lifestyle choices, and socioeconomic factors.

In conclusion, the promotion of global partnerships and information sharing can aid in the implementation of optimal methodologies and inventive strategies for the treatment of thyroid disorders. The reach of healthcare can be increased by integrating telemedicine and digital health technologies, especially in rural areas. Bangladesh may take major steps toward managing thyroid diseases, advancing global health goals, and guaranteeing a healthy future for its people by implementing these recommendations and promoting a multifaceted approach.

## 6 Limitations of the Study

Our study has some limitations. First, the survey relies on self‐reported information from respondents regarding their thyroid disorders, family history, and comorbidities. Self‐reporting can introduce recall bias, where participants may not accurately remember or report their medical history or symptoms. Second, the study is conducted only in the southeastern part of Bangladesh. This limits the generalizability of findings to other regions within Bangladesh or other Southeast Asian populations where environmental, dietary, genetic, and healthcare access factors might differ. Addressing these limitations could strengthen the validity and applicability of the survey's findings, providing more robust insights into thyroid disorders within the Bangladeshi population and potentially informing better healthcare strategies and policies.

## Author Contributions

**Muhammad Shahadat Hossen:** conceptualization, investigation, methodology. **Md. Mahmodul Islam:** visualization, methodology, writing–original draft. **Abhijit Das:** formal analysis, data curation. **Md. Abdur Rahman Ripon:** writing–original draft, data curation. **Mohammad Tohidul Amin:** formal analysis, writing–review and editing. **Mohammad Anwarul Basher:** writing–review and editing, methodology. **Md. Mamun Or Rashid:** conceptualization, supervision, writing–review and editing.

## Conflicts of Interest

All authors have read and approved the final version of the manuscript. The corresponding author had full access to all of the data in this study and takes complete responsibility for the integrity of the data and the accuracy of the data analysis. The authors confirm that we have no conflicts of interest in this work.

## Transparency Statement

The lead author Md. Mamun Or Rashid affirms that this manuscript is an honest, accurate, and transparent account of the study being reported; that no important aspects of the study have been omitted; and that any discrepancies from the study as planned (and, if relevant, registered) have been explained.

## Supporting information

Supporting information.

## Data Availability

The datasets are available from the corresponding author upon reasonable request. Supplementary data (Supporting Information S1: Table S1) is attached here.
