# Supplementary material for: Thyroid Dysfunction Prevalence and Risk Factors in the Southeastern Part of Bangladesh: A Cross‐Sectional Study
Source: Health Sci Rep. 2025 Jan 7;8(1):e70329. doi: 10.1002/hsr2.70329 (PMC11705530; doi:10.1002/hsr2.70329)
Supplement: Supplementary file 1 — Supporting information. [file HSR2-8-e70329-s001.docx]

**Supplementary Table S1:** Survey Questionary

| **Questions** |
| --- |
| 1. Which kind of thyroid disorder are you suffering from? |
| 1. What kind of symptoms have you felt during thyroid disease? |
| 1. Since when are you suffering from thyroid disease? |
| 1. Is this a genetical disease in your family? |
| 1. Is there any problem for having pregnant/conceive? (In case of female) |
| 1. Have you met any gynecologist during this disease? (In case of female) |
| 1. Do you have any other diseases associated with this disease? |
| 1. Which dosage form/amount are you using in the treatment of thyroid disorder? |
| 1. What kind of food habit do you have? |
| 1. What are the results of hormonal tests from patient’s documents about TSH, FT_4_, FT_3_, T_4_ and T_3_ level? |
